# Deciphering the role of interleukin-22 in metabolic alterations

**DOI:** 10.1186/s13578-015-0060-8

**Published:** 2015-12-15

**Authors:** Robert Sabat, Kerstin Wolk

**Affiliations:** Psoriasis Research and Treatment Center, University Hospital Charité, Charitéplatz 1, 10117 Berlin, Germany; Research Center Immunosciences, University Hospital Charité, Charitéplatz 1, 10117 Berlin, Germany; Interdisciplinary Group of Molecular Immunopathology, Dermatology/Medical Immunology, University Hospital Charité, Charitéplatz 1, 10117 Berlin, Germany

**Keywords:** Obesity, Metabolic alterations, Immunity, Th1-cells, Th17-cells, Th22-cells, ILC3, Diabetes mellitus, Arteriosclerosis, IL-17, TNF-α

## Abstract

Inflammatory processes and metabolic alterations are supposed to substantially interact. Recently, cumulating reports describe a profound role of interleukin(IL)-22 in this relationship. IL-22 is a particular kind of immune mediator that is produced by certain lymphocyte populations and regulates the function of several tissue cells but not immune cells. So far, IL-22 was known to plays a fundamental role in the elimination of bacterial infections at border surfaces of the body and to protect tissues from damage. This research highlight article arranges the facts regarding the effects of IL-22 in the context of adiposity and metabolic alterations and postulates a new function of the immune system.

## The IL-22/IL-22R1 system

**Fig. 1:**
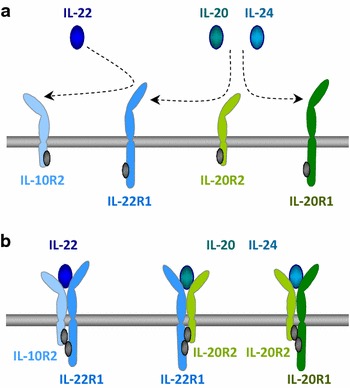
IL-22, IL-20, and IL-24 and their receptor complexes. Binding of IL-22, IL-20, and IL-24 to their receptor complexes takes place in two steps: IL-22 initially binds to IL-22R1 while IL-20 and IL-24 primarily interact with IL-20R2 (**a**). The complete receptor complex for IL-22 consists of IL-22R1 and IL-10R2. IL-20 and IL-24 can use two different receptor complexes: the IL-22R1/IL-20R2 complex and the IL-20R1/IL-20R2 complex (**b**)

IL-22 is a small protein discovered in 2000 by the research group of Renauld [1]. The main producers of IL-22 are CD4+ effector/memory T-cells including T-helper(Th)1-, Th17-, and Th22-cells as well as group 3 innate lymphoid cell (ILC3), the latter comprising natural killer cells, lymphoid tissue inducer like cells, and natural cytotoxicity receptor-positive ILCs [1]. The exact cellular sources of IL-22 in human diseases are often unknown and probably vary depending on the nature of the disorders. While the IL-22 secretion by Th-cells occurs following specific antigenic stimulation supported by antigen-presenting cells, IL-22 production by ILC3 is provoked by cytokines like IL-23, IL-1β, and tumor necrosis factor-α (TNF-α). This is important, because low inflammation is observed in people with adiposity and metabolic alterations and, via these inflammatory mediators, might cause permanent IL-22 production. IL-22 acts on cells situated near its producers. Additionally, it reaches the bloodstream, where it is probably stabilized by IL-22-binding protein, and can therefore also influence cells far from its production site [1]. To mediate its biological effects, IL-22 uses a receptor complex composed of two different transmembrane chains: IL-22R1 and IL-10R2 (Fig. 1) [1]. IL-10R2 additionally communicates the presence of IL-10, IL-26, IL-28, and IL-29 and is carried by many different types of cells. As we demonstrated already in 2004, IL-22R1 determines the cellular sensitivity towards IL-22, is restricted to specific cell types, and is lacking on immune cells [2]. Accordingly, IL-22 represents a novel type of immune mediators that, although produced by immune cells, regulates the function of quite few tissue cells. The cells carrying IL-22R1 comprise those of epithelial origins as well as fibroblast. Interestingly, IL-22R1 (in a complex with IL-20R2) is also used by the IL-22 “relatives” IL-20 and IL-24 (Fig. 1) [1]. Organs with strong IL-22R1 expression include the skin, liver, kidney, pancreas, and those of the respiratory and gastrointestinal systems [2]. IL-22 enhances the innate immunity of epithelia and plays a fundamental role in the elimination of bacterial infections at body surfaces [2]. Furthermore and as first demonstrated for hepatocytes/the liver by the research group of Bin Gao, IL-22 protects IL-22R1-carrying cells from damage [3]. Such protection was later confirmed for pancreas and kidney. Although primarily protective, the very same effects might underlie the pathogenic role of IL-22 in some inflammatory diseases, as previously shown for psoriasis [2]. This is especially evident, when IL-22 effects are modulated and/or amplified by other cytokines such as IL-17 and/or TNF-α. In addition to all these data, recent reports announce a completely new kind of IL-22 action, which this article will focus on: as an intermediary between inflammation and metabolic alterations. In this context, there are two important questions: (1) Does endogenous IL-22 modulate the development of adiposity and metabolic alterations? And (2), can exogenous IL-22 be used for the therapeutic treatment of adiposity and metabolic alterations?

## Role of endogenous IL-22 in the development of adiposity and metabolic alterations

**Fig. 2:**
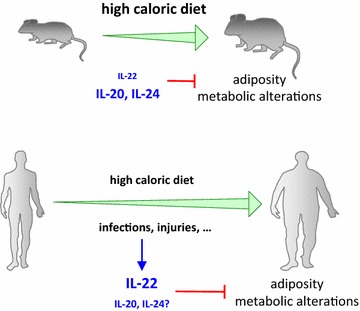
Role of endogenous IL-22 in the development of adiposity and metabolic alterations in mice and men. Adiposity can be induced by high caloric diet in mice and humans. Respective mouse models are based on massively forced fat intake of animals kept under pathogen-free conditions and usually involves very young mice (they are typically analyzed at an age of 20 % of their maximum age). Using such models, IL-22-deficient mice do not show a specific phenotype, although the activation of IL-22R1 by endogenous ligands seems to moderately limit the development of adiposity and metabolic alterations. In contrast to these mouse models, the development of adiposity and metabolic alterations in humans usually takes long time periods and leads to death within the second half of life. Importantly, banal infections or injury that occur over the extended time period might amplify the generation of IL-22-producing cells. We postulate that this endogenous IL-22 is a new counter-mechanism of the immune system to limit the development of adiposity and metabolic alterations

The development of adiposity can be nicely investigated in animal models generated by feeding of mice with high-fat diet (HFD) for several weeks. As independently demonstrated by Wenjun Ouyang’s and Bin Gao’s groups, however, endogenous IL-22 does not play any role in the development of adiposity and metabolic alterations in these mice [4, 5]. In fact, there were no differences in body weight, glucose tolerance, and insulin resistance between IL-22-deficient mice and wild-type littermate controls. The reason for this lacking phenotype might lie in the very low levels of IL-22 (comparable to those in control mice) in young mice fed with high caloric diet and bred without other sources of inflammation. However, it seems that we cannot simply transfer these animal-based observations to the human situation [5]. The frequency of IL-22-producing Th-cells in blood of patients suffering from type 2 diabetes mellitus (DM2) are higher than in control individuals [6]. Furthermore, IL-22-producing CD4+ T cells are enriched in adipose tissue of such patients [7, 8]. IL-1β, known for a long time as being strongly present in white fat tissue of adipose people, promotes IL-22 production by human adipose tissue CD4+ T cells [8]. Accordingly, blood concentration of IL-22 is elevated in DM2 patients [7]. The reason for this difference to mice might be that most human subjects with adiposity and metabolic alterations are older, their development of adiposity and metabolic alterations takes longer, and during this time these people sometimes have externally triggered inflammation (e.g. caused by banal infection) that might amplify the generation of lL-22-producing cells. We postulate that the presence of elevated IL-22 levels in adipose people is a counter-mechanism of the immune system to limit the development of adiposity and metabolic alterations (Fig. 2). This hypothesis contrasts the current notion that inflammation generally worsens metabolic alterations. Hints for such counter-mechanism by inflammatory mediators come from studies with IL-22R1-deficient mice. In fact, these animals were fatter after already the first month of HFD ([4] and own observation) and developed higher levels of glucose intolerance and insulin resistance within 3 months [4]. This suggests that, already in quite young animals, endogenous IL-22R1 ligands—in this case other than IL-22 (i.e., IL-20 and/or IL-24)—are indeed able to counteract the development of adiposity (Fig. 2).

## IL-22 as therapeutic agent for adiposity and metabolic alterations

**Fig. 3:**
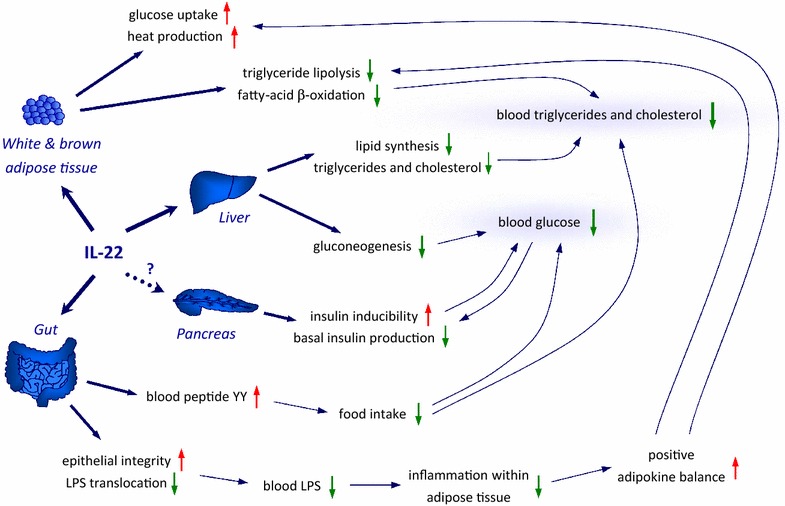
Potential effects of exogenous IL-22 that may play a role in the reduction of adiposity and metabolic alterations in humans

A recent report from *Cell and Bioscience* as well as three other manuscripts described that systemic application of exogenous IL-22 is able to improve metabolic alterations in obese mice [4, 5, 9, 10]. The efficacy of such treatment seems to be dependent on both the dose of applied IL-22 and the previous duration of the metabolic disease. In fact, high IL-22 levels (obtained e.g. by intraperitoneal application of 2 μg/g mouse of IL-22Fc protein twice a week) markedly reduced body weight, decreased blood glucose levels under both fed and fasting conditions and alleviated glucose intolerance and insulin resistance in mice that had been fed with HFD for 8 weeks [4]. In contrast, twice-a-week administration of 20 ng/g mouse of rmIL-22 did not affect body weight, fasting glucose levels, or insulin resistance in mice fed with HFD for previous 5 months [5]. Importantly, counteraction of metabolic alterations with elevated IL-22 levels seems to be based on numerous different effects of this cytokine (Fig. 3). First, IL-22Fc can raise serum levels of peptide YY, an anorexic gut hormone regulating food intake, and indeed minimizes food ingestion in young adipose mice [4]. Furthermore, IL-22 enhances the gut epithelial integrity and decreases the translocation of bacterial products like lipopolysaccharide (LPS) into blood serum. Thereby, it diminishes the LPS blood concentration, and thus indirectly reduces the activation of macrophages in adipose tissue [4]. The most prominent effects of IL-22 in this context, however, are probable those on hepatocytes that result in the increase of liver functionality. Long-term treatment with rmIL-22 decreased the hepatic expression of enzymes for lipid synthesis like ATP citrate lyase (ACLY) as well as elongation of very long chain fatty acids like 6 (ELOVL6) and reduced hepatic triglyceride and cholesterol levels [9]. Furthermore, it decreased the expression of enzymes for gluconeogenesis like glucose-6-phosphatase and phosphoenolpyruvate carboxykinase (PEPCK) in hepatocytes and suppressed glucose production [5]. Additionally, high IL-22 levels were shown to act on adipocytes and elevate the expression of genes involved in triglyceride lipolysis [e.g. hormone-sensitive lipase (LIPE) and patatin-like phospholipase domain-containing protein 2 (PNPLA2)] and fatty-acid β-oxidation (acyl-CoA oxidase 1, ACOX1) [4]. Accordingly, the triglyceride content in white adipose tissue and serum was reduced in adipose mice treated with IL-22Fc construct [4]. High IL-22 levels seem to increase the insulin sensitivity of the liver, muscle, and adipose tissue. Accordingly, IL-22 appears to increase glucose uptake in brown adipose tissue and heat production [4, 10]. Finally, IL-22 treatment leads to reduction of constitutive serum insulin concentration in obese mice and restores insulin production in response to glucose provocation [4, 5, 10]. Whether the last fact is co-determinated by a direct effect of IL-22 on pancreatic beta-cells is currently controversially discussed [5, 10]. While some reports do not observe IL-22R1 expression and IL-22-induced signal transduction in beta-cells [5], others postulated that IL-22 suppresses oxidative and endoplasmic reticulum stress in these cells and facilitates secretion of high-quality efficacious insulin [10]. Regardless of this controversy, it remains clear, that systemic application of exogenous IL-22 is able to improve metabolic alterations in adipose mice. It is currently impossible to estimate the extent we can use these very interesting discoveries for the development of the innovative treatment of human adiposity and disturbed metabolism. Not only because experiments with hepatocytes or adipocytes from obese subjects regarding IL-22 effects are missing so far. In principal, it appears quite easy to reach elevated IL-22 levels in humans: This can be achieved by application of this cytokine itself or by the activation of IL-22-inducing key signal transduction elements (e.g. aryl hydrocarbon receptor stimulation) by small molecules [as already shown for 6-formylindolo(3, 2-b)carbazole] [1]. Further studies need to clarify whether levels of IL-22 reached in this way will be sufficient and safe. So far, at least the concerns regarding the safety are rather limited due to the restricted target cell range of IL-22, especially its lacking immune modulation.

## Abbreviations

IL: interleukin
Th: T helper
R: receptor
ILC3: group 3 innate lymphoid cell
HFD: high fat diet
DM2: type 2 diabetes mellitus
LPS: lipopolysaccharide
